# Genetic diversity of clinical isolates of *Bacillus cereus *using multilocus sequence typing

**DOI:** 10.1186/1471-2180-8-191

**Published:** 2008-11-06

**Authors:** Alex R Hoffmaster, Ryan T Novak, Chung K Marston, Jay E Gee, Leta Helsel, James M Pruckler, Patricia P Wilkins

**Affiliations:** 1 National Center for Zoonotic, Vector-borne, and Enteric Diseases, Centers for Disease Control and Prevention, Atlanta, USA

## Abstract

**Background:**

*Bacillus cereus *is most commonly associated with foodborne illness (diarrheal and emetic) but is also an opportunistic pathogen that can cause severe and fatal infections. Several multilocus sequence typing (MLST) schemes have recently been developed to genotype *B. cereus *and analysis has suggested a clonal or weakly clonal population structure for *B. cereus *and its close relatives *B. anthracis *and *B. thuringiensis*. In this study we used MLST to determine if *B. cereus *isolates associated with illnesses of varying severity (e.g., severe, systemic vs. gastrointestinal (GI) illness) were clonal or formed clonal complexes.

**Results:**

A retrospective analysis of 55 clinical *B. cereus *isolates submitted to the Centers for Disease Control and Prevention between 1954 and 2004 was conducted. Clinical isolates from severe infections (n = 27), gastrointestinal (GI) illness (n = 18), and associated isolates from food (n = 10) were selected for analysis using MLST. The 55 isolates were diverse and comprised 38 sequence types (ST) in two distinct clades. Of the 27 isolates associated with serious illness, 13 clustered in clade 1 while 14 were in clade 2. Isolates associated with GI illness were also found throughout clades 1 and 2, while no isolates in this study belonged to clade 3. All the isolates from this study belonging to the clade 1/cereus III lineage were associated with severe disease while isolates belonging to clade1/cereus II contained isolates primarily associated with severe disease and emetic illness. Only three STs were observed more than once for epidemiologically distinct isolates.

**Conclusion:**

STs of clinical *B. cereus *isolates were phylogenetically diverse and distributed among two of three previously described clades. Greater numbers of strains will need to be analyzed to confirm if specific lineages or clonal complexes are more likely to contain clinical isolates or be associated with specific illness, similar to *B. anthracis *and emetic *B. cereus *isolates.

## Background

The phylogenetically related species of the *Bacillus cereus *group include: *B. cereus*, *B. anthracis*, *B. thuringiensis*, *B. mycoides *and two recently described species, *B. pseudomycoides *and *B. weihenstephanensis *[1-4]. *B. cereus, B. thuringiensis*, and *B. anthracis *have been the most characterized due to their pathogenic nature. *B. anthracis *is the etiologic agent of anthrax, *B. thuringiensis *is an insect pathogen, and *B. cereus *can be associated with a variety of human infections. *B. cereus *is most frequently associated with food poisoning, characterized by strains producing emetic or diarrheal toxins. It is also an opportunistic pathogen resulting in localized (wound, ocular, oral) and systemic infections (bacteremia, septicemia, endocarditis, meningitis, pneumonia) especially in immunocompromised patients [5,6]. However, although *B. cereus *is ubiquitous in the environment, particularly soil, it does not generally pose a health threat.

Systemic infection due to *B. cereus *can be associated with significant morbidity and mortality. Typically the species of *Bacillus *most commonly associated with serious human illness is *B. anthracis*. However, pulmonary infections caused by *B. cereus *can be severe and fatal but have been described predominantly in patients with significant risk factors [7-14]. There are rare reports in the literature of fulminating pneumonia associated with *B. cereus *infections in patients not known to be immune compromised [15-18]. Interestingly, *B. cereus *strains have been shown to harbor diverse plasmids which can share conserved sequences with *B. anthracis *virulence plasmids pXO1 and pXO2 [19-21], and isolates associated with a few of these severe infections were shown to contain *B. anthracis *virulence genes. *B. cereus *G9241, an isolate associated with a severe pneumonia case, contained a plasmid, pBCXO1, which was 99.6% similar to the *B. anthracis *pXO1 virulence plasmid [17]. More recently several *B. cereus *isolates, associated with fatal pneumonias in Texas, were described that harbored *B. anthracis *toxin and/or capsule virulence genes (i.e., *pagA*, *lef*, *cya*, and *capBCA*) [17,22]. G9241 and the Texas isolates were recovered from patients who were not obviously immunocompromised; however, all three infections were in metal workers, an occupation that may have affected their susceptibility to respiratory infections [23-25].

The population structure of pathogenic bacteria varies over a wide range, from strictly clonal to effectively panmictic [26]. A study of localized populations of the *B. cereus *group in soil using multilocus sequence typing (MLST) suggested frequent recombination among *B. weihenstephanensis*, while *B. cereus *and *B. thuringiensis *appeared clonal [27]. Two additional MLST studies, each using different MLST gene sets, also suggested a weakly clonal to clonal *B. cereus *population structure with evolutionary changes occurring through point mutations [4,28].

While the overall population structure of the *B. cereus *group is not yet clear due to limited numbers of strains examined, data suggest that in addition to *B. anthracis*, some pathogenic *B. cereus *isolates associated with periodontal disease and emetic gastrointestinal illness are limited to specific clonal groups [29,30]. A recent MLST study found 30 of 48 isolates of clinical origin were associated with clonal complexes and some groups contained strains isolated from similar human sources [31]. In contrast, two studies using MLST analysis on smaller numbers (n = 8 and n = 10) of *B. cereus *isolates from severe infections were found to be diverse and not restricted to single clonal groups or lineages [3,32]. In this study we used MLST to examine the phylogenetic diversity and relatedness of 55 clinical *B. cereus *isolates, from a historical collection of *B. cereus *recovered from 1954–2004.

## Methods

### Bacterial isolates

A total of 55 strains of *B. cereus *associated with severe (septicemia and pneumonia) or gastrointestinal illness were analyzed (Table 1 and 2). The strains were selected from over 400 *Bacillus *in the collection of the Special Bacteriology Reference Laboratory, Centers for Disease Control and Prevention. The isolates represent a diverse collection of strains received between 1954 and 2004 from 19 states in the U.S. All isolates found in the collection associated with severe systemic human infections of the blood or lungs were selected for this study. This resulted in the identification of 27 isolates from 26 epidemiologically distinct infections. In addition, a convenient sampling of 28 isolates associated with cases of GI illness or food remnants connected to eight foodborne outbreaks and five sporadic cases of food poisoning were included for comparison. Foodborne isolates were generally selected if associated with two or more clustered cases. All strains shared common biochemical/phenotypic characteristics that are consistent with the identification of *Bacillus cereus*. They were motile rods with peritrichous flagella, and hemolytic with β-hemolysis or lavender-green coloration under heavy growth indicating proteolysis. They produced catalase, lecithinase, and reduced nitrate. They fermented D-glucose and maltose but were variable in fermenting lactose and sucrose. There was no fermentation of D-xlyose and D-mannitol. Production of oxidase and urease was variable while there was no production of indole. Toxin crystals were not detected for these isolates. 16S DNA sequencing was determined as described previously [33]. Isolates were cultured on trypticase soy agar containing 5% sheep blood (Becton Dickinson Microbiology Systems, Sparks, MD) at 37°C overnight. Bacterial genomic DNA extractions for PCR were prepared as described previously [34]. Emetic isolates were identified by PCR as described by Ehling-Shulz et al. [35].

**Table 1:** Characteristics of isolates associated with severe disease

**ID**	**ST**^a^	**Origin**^b^	**Source**	**Year isolated**	**Clade**^c^	**Additional information**
03BB102^d^	11	TX	Blood	2003	1	Pneumonia, fatal
F4976	24	OR	Lung	1983	2	Pneumonia
E6345	26	NH	Lung	1979	1	
D4214	62	TX	Blood	1975	1	
B5780	76	TX	Blood	1970	1	
G9241^e^	78	LA	Sputum	1994	1	Pneumonia
G9898^f^	78	LA	Sputum	1996	1	Pneumonia, fatal
03BB87^d^	78	TX	Blood	2003	1	Pneumonia, fatal
04S 00334	85	CA	Blood	2004	2	
B4266	89	VA	Lung	1969	2	Pneumonia, fatal
H1548	90	NC	Blood	2000	1	Septicemia, fatal
B1357	91	LA	Lung	1968	1	
F9930	93	GA	Lung	1987	2	Biopsy
F3920	94	CA	Lung	1982	2	Pneumonia
G1440	94	LA	Lung	1988	2	Pneumonia, biopsy
G4200	95	MS	Blood	1990	2	Pneumonia, fatal
D8096	100	LA	Blood	1971	2	
1952	101	GA	Blood	1955	2	Septicemia, fatal
E3911	102	TX	Blood	1978	1	
F4794	111	MI	Blood	1982	2	Pneumonia
G3317	121	Israel	Blood	1989	1	
F5191^g^	122	CA	Lung	1983	1	Biopsy
C3276	136	TX	Blood	1972	2	
E1149	138	MO	Lung	1978	2	
F5190^g^	140	CA	Lung	1983	1	Biopsy
2003-I-1253	142	OR	Blood	2003	2	Fatal
SB460	146	NY	Blood	1987	2	Septicemia

^a ^ST = sequence type

^b ^Country or U.S.A. state abbreviation

^c ^Clade as described by Priest et al. [28]

^d ^Hoffmaster et al. [22]

^e ^Hoffmaster et al. [17]

^f ^Sue et al. [42]

^g ^Distinct isolates from same patient biopsy

**Table 2:** Characteristics of GI-associated isolates

**ID**	**ST**^a^	**Origin**^b^	**Source**	**Year isolated**	**Clade**^c^	**Addtional information**
F6722	4	AL	Food	1985	2	Food poisoning, diarrheal toxin
F663^d^	26	NC	Food	1981	1	Outbreak, emetic toxin^k^
F665^d^	26	NC	Stool	1981	1	Outbreak, emetic toxin^k^
F667^d^	26	NC	Stool	1981	1	Outbreak, emetic toxin^k^
H3074.97^e^	26	TX	Stool	1997	1	Outbreak, emetic toxin^k^
H2573.97^f^	26	England	Food	1997	1	Outbreak, emetic toxin^k^
H2576.97^f^	26	England	Food	1997	1	Outbreak, emetic toxin^k^
H9310	26	MN	Vomit	2000	1	Outbreak, emetic toxin^k^
H9311	26	MN	Food	2000	1	Outbreak, emetic toxin^k^
H9312	26	MN	Vomit	2000	1	Outbreak, emetic toxin^k^
G9844^g^	56	NE	Stool	1996	2	Outbreak
G9842^g^	56	NE	Stool	1996	2	Outbreak, diarrheal toxin
G9843^g^	56	NE	Stool	1996	2	Outbreak
G2008^e^	73	PA	Stool	1988	2	Food Poisoning
F666^d^	92	NC	Stool	1981	1	Outbreak
C1784^i^	96	FL	Food	1972	2	Diarrhea
C1617	97	NC	Stool	1972	2	
F439^j^	98	MI	Stool	1981	2	Diarrhea, diarrheal toxin
F438^j^	98	MI	Stool	1981	2	Diarrhea, diarrheal toxin
G2055^h^	99	PA	Stool	1988	2	Food poisoning, diarrheal toxin
G2054^h^	120	PA	Food	1988	1	Food poisoning, diarrheal toxin
C1785^i^	123	FL	Food	1972	1	Diarrhea
C1783^i^	123	FL	Stool	1972	1	Diarrhea
3297	124	AZ	Stool	1957	2	Food poisoning
F5157	137	CA	Food	1983	2	Food poisoning, diarrheal toxin
F7720	141	NM	Stool	1986	1	Diarrheal toxin
H3076.97^e^	144	TX	Stool	1997	1	Outbreak, emetic toxin^k^
H3081.97^e^	144	TX	Food	1997	1	Outbreak, emetic toxin^k^

^a ^ST = sequence type

^b ^Country or U.S.A. state abbreviation

^c ^Clade as described by Priest et al. [28]

^d ^North Carolina 1981 food outbreak, 18 people, linked to rice

^e ^Texas 1997 food outbreak, linked to rice

^f ^England 1997 food outbreak, linked to rice

^g ^Nebraska food outbreak, three people, source unknown

^h ^Food poisoning, one person, source unknown

^i ^Food poisoning linked to one of two food sources tested

^j ^Different patients, cases linked

^k ^Presence of emetic toxin genetic determinants were detected by PCR [35].

### MLST

The isolates were characterized by the MLST scheme described by Priest et al. [28] and performed with modifications described previously [36]. Briefly, approximately 10 ng of DNA extracted from bacterial cultures were used as the template in the PCR reactions with the primers described on the *B. cereus *MLST website  to generate amplicons from the seven MLST gene loci (*glpF, gmk, ilvD, pta, pur, pycA *and *tpi*). Primers described for "Option 1" were used in the cases of the *ilvD *and *gmk *gene primers. Amplification products of the correct size and similar concentrations, based on visual inspection of agarose gels, were obtained from all 55 isolates and were then purified by using a QiaAmp PCR purification kit (Qiagen Inc., Valencia, CA) according to the instructions of the manufacturer.

The nucleotide sequences of both DNA strands of the PCR fragments were determined using the amplification primers and the BigDye terminator cycle sequencing kit version 3.1 according to the manufacturer's instructions, except 3 μl of BigDye was used instead of 8 μl (Applied BioSystems, Foster City, CA). Sequencing products were separated from unincorporated dye terminators by Centri-Sep column purification (Princeton Separations, Adelphia, NJ) and resolved with an Applied Biosystems model 3100 automated DNA sequencing system.

### Data analysis

Sequence editing and alignment was completed using the Genetics Computer Group Wisconsin Package version 10.3 (Accelrys, San Diego, CA). The chromatograms from the ABI 3100 sequencer were exported, visually examined, and assembled in SEQMERGE. The sequences of each of the seven housekeeping genes were edited to the previously described allele lengths (between 348 and 504 bp). These were then assigned allele numbers based on those already described in the *B. cereus *MLST database . Isolates were assigned a sequence type (ST) on the basis of the combination of the seven alleles. Allelic sequences differing from known alleles were assigned new allele numbers and ST and added to the *B. cereus *MLST database.

The seven gene fragments from each of the 55 isolates were concatenated and downloaded from the MLST website. To assess the relationships of the isolates in this study with other strains, a phylogenetic tree was derived from the aligned concatenated sequences using the neighbor-joining method [37] using the Kimura 2-parameter model [38] and 1000 step bootstrap analysis of the data using Mega 3 [39], as described previously [36]. Simpson's index of diversity was calculated as described by Hunter and Gaston [40].

## Results

Forty-five clinical and 10 food-associated isolates of *B. cereus *were selected from over 400 *Bacillus *submitted to the CDC Special Bacteriology Reference Laboratory between 1954 and 2004 and were analyzed using MLST. Twenty-seven strains were recovered from severe atypical *Bacillus *infections in 26 patients presenting with either pneumonia or septicemia (Table 1). Eight of these 26 cases were fatal. Twenty-eight isolates were recovered from sporadic cases of GI illness, foodborne outbreak investigations or associated foods. The 55 isolates represented 38 STs (Table 3), 27 of which were new to the MLST database . The number of alleles per locus observed in the isolates in this study ranged from 18–23 and averaged 19.9. The Simpson's index of diversity was calculated using a subset of epidemiologically distinct isolates and found to be 0.989 (where 1.0 is equal to absolute discrimination).

**Table 3:** Sequence types and alleles grouped by lineage

**ST**^a^	**N**^b^	**glpF**	**gmk**	**ilvD**	**pta**	**pur**	**pycA**	**tpi**	**Clade/lineage**^c^
90	1	6	4	41	5	43	46	3	1/Cereus I
92	1	6	4	42	4	16	6	3	1/Cereus I
120	1	6	4	3	4	46	6	36	1/Cereus I
144	1	67	2	63	5	36	3	4	1
26	4	3	2	31	5	16	3	4	1/Cereus II
140	1	52	2	21	5	19	3	38	1/Cereus II
91	1	51	2	21	5	19	3	38	1/Cereus II
141	1	54	2	21	5	19	3	4	1/Cereus II
121	1	53	2	59	54	47	3	2	1/Cereus II
122	1	53	2	59	17	47	3	2	1/Cereus II
11	1	34	1	32	1	33	37	24	1/Cereus III
62	1	38	1	32	1	18	33	24	1/Cereus III
76	1	34	15	28	38	30	29	33	1/Cereus III
78	3	24	22	33	37	34	38	5	1/Cereus IV
123	1	48	30	33	37	44	48	45	1/Cereus IV
101	1	33	33	44	19	2	17	17	2/Toworthi
96	1	33	8	44	19	2	17	17	2/Toworthi
89	1	14	8	40	19	2	17	17	2/Toworthi
94	2	13	29	9	14	9	12	31	2/Toworthi
73	1	13	8	9	14	9	12	31	2/Toworthi
99	1	13	8	9	14	46	12	31	2/Toworthi
24	1	12	8	9	14	11	12	10	2/Toworthi
100	1	13	8	46	28	9	12	7	2/Toworthi
142	1	13	8	8	11	9	12	7	2/Toworthi
4	1	13	8	8	11	11	12	7	2/Toworthi
95	1	13	29	9	11	2	12	8	2/Toworthi
93	1	14	29	43	11	9	47	8	2/Toworthi
98	1	40	28	14	12	2	50	7	2/Kurstaki
85	1	47	28	14	12	2	36	7	2/Kurstaki
137	1	37	28	14	12	37	36	7	2/Kurstaki
136	1	31	8	14	44	2	36	7	2/Kurstaki
138	1	37	9	14	12	12	14	7	2/Kurstaki
124	1	14	32	48	45	58	51	7	2/Kurstaki
146	1	68	20	23	8	4	8	39	2/Thuringiensis
97	1	15	6	47	8	4	8	7	2/Thuringiensis
56	1	15	7	7	2	7	26	13	2/Sotto
111	1	43	26	35	42	39	41	30	2
102	1	49	31	45	43	45	49	37	1

^a ^ST = sequence type

^b ^Number of isolates identified from epidemiologically distinct (unrelated) events.

^c ^Clade as described by Priest et al. [28]

**Figure 1:**
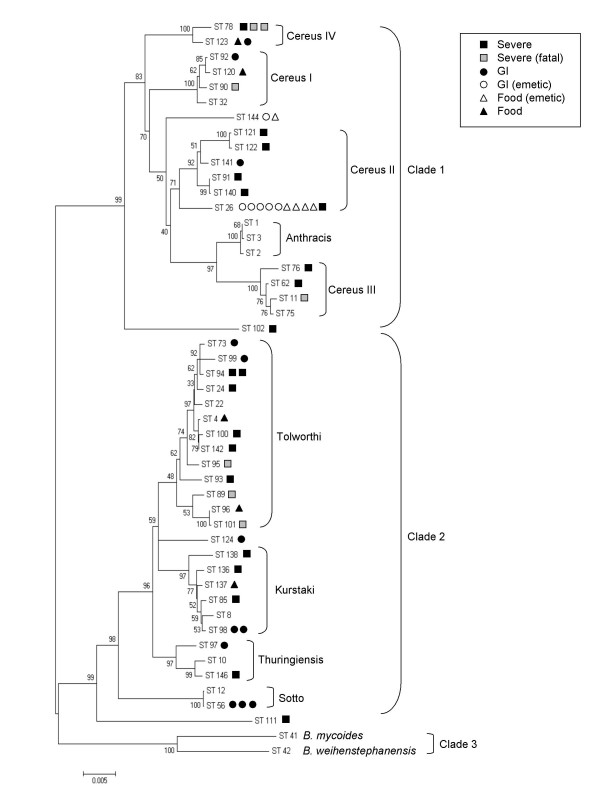
**Relationships between isolates of this study and other select reference isolates using concatenated sequences from seven housekeeping alleles.** Tree was constructed using the neighbor joining method and percent bootstrap confidence levels were calculated using 1000 resamplings of the original data. Clades and lineages are labeled as designated by Priest et al. [28] with the addition of Cereus IV.

A neighbor-joining tree was constructed using the seven concatenated allele sequences from the isolates in this study and selected isolates from the MLST database (Figure 1). The isolates in this study were not monophyletic by morbidity or mortality; instead, they were distributed among the *Bacillus *group clades 1 and 2 and in numerous lineages as previously defined [28].

### Clade 1

Thirteen of 27 isolates associated with severe illness grouped in clade 1, while nine of 18 GI-associated isolates and seven of 10 food isolates were also grouped to this clade. Isolates associated with severe illness were found in all three previously described Cereus lineages within clade 1. Isolates recovered from GI illness grouped in Cereus I and II lineages, but not Cereus III.

All of the emetic *B. cereus *isolates were represented by two (ST 26 and 144) of the four previously described STs of emetic strains [41]. ST 26 was the most commonly identified ST in this and other reports [28,41]. Over half (6/9) of the GI-associated clinical isolates and five of the seven food isolates in clade 1 were associated with emetic GI illness (Figure 1).

Three STs (ST 102, 78 and 123) found to cluster in clade 1 did not group into described lineages [28]. The ST 78 and 123 isolates were sufficiently divergent to possibly warrant a designation of a fourth Cereus lineage (Cereus IV) in clade 1 (Figure 1). All three isolates of ST 78 were associated with severe pneumonia cases, of which two were fatal. Both isolates of ST 123 were associated with a diarrheal disease and group with ST 78 isolates, based on alignment of the concatenated sequences, however, these two STs share only two of seven allele types (Table 3).

### Clade 2

Fourteen of 27 isolates associated with severe illness grouped in clade 2, while nine of 18 GI-associated isolates and three of 10 food isolates were also grouped into this clade. Isolates recovered from severe illness were found in three of four previously described Cereus lineages within clade 2, but not in the Sotto lineage [28]. Isolates associated with GI illness grouped in all four clade 2 lineages (Figure 1). A single isolate associated with a pneumonia case (ST 111) clustered to clade 2, but did not belong to any of the described lineages.

## Discussion

In this study we used MLST to examine the phylogenetic diversity and relatedness of *B. cereus *isolates that were associated with different clinical presentations. Allelic diversity among the isolates in the study was examined and the average number of alleles per locus was determined to be 19.9. Previously, Helgason et al. found an average of 33.6 alleles per locus in 67 *Bacillus *isolates [4] and Priest et al. found an average of 30.5 alleles per locus in 105 isolates [28]. Adjusted for the number of isolates included in the study, the allelic diversity in this study was less than that described by Helgason and greater than the diversity reported by Priest et al. (0.5 > this study-0.36 > Priest-0.29). We used the Simpson's index of diversity to measure the discriminatory power of this subtyping method (i.e., likelihood of two isolates from epidemiologically distinct events having the same ST). The overall discrimination index was 0.989 (where 1.0 is equal to absolute discrimination), making MLST a very useful tool for molecular epidemiology. However, this value is clearly biased by the types of isolates that are included. For instance, the discrimination would be quite poor for analysis of foodborne isolates associated with emetic disease (for which only four STs have been observed).

Only three STs were seen more than once from epidemiologically distinct cases (ST 26, 78 and 94). ST 26 was associated with four separate emetic GI outbreaks. This was not unexpected since ST 26 has been the most common ST associated with emetic isolates in previous studies [28,41]. ST 78 and ST 94 are not closely related (i.e., ST 78 is in clade 1 and ST 94 is in clade 2) however, all the isolates of these STs (n = 5) were associated with pneumonia cases. Interestingly, all three of the ST 78 isolates were the cause of pneumonia in metal workers (two cases were fatal). In addition, we have previously shown all three isolates (G9241, G9898, and 03BB87) produce a capsule and were PCR positive for pBC218 plasmid genes (putative polysaccharide polymerase and translocase genes) hypothesized to be required for a polysaccharide capsule production in G9241 [17,22,42]. Thus far, we have only seen these plasmid genes in isolates of ST 78. Perhaps this is an under sampled clonal group harboring a specific virulence plasmid similar to *B. anthracis*.

The clade 1/Cereus III lineage is most closely related to the Anthracis lineage and was previously shown to include both *B. cereus *and *B. thuringiensis *[28]. All the Cereus III isolates analyzed for this study were associated with systemic illness. This lineage contained isolates from Texas, including 03BB102 (ST 11) which was isolated from a fatal case of pneumonia in a welder [22], and a ST 62 clinical isolate recovered from a septicemic patient (TX, 1975). The other isolate in this lineage, B5780, was recovered from blood and shares ST 76 with another invasive isolate in the MLST database.

The clade 1/Cereus II lineage, which is also closely related to the Anthracis/Cereus III lineages, contained isolates from both systemic and GI illnesses. Nine of the 10 GI-associated isolates, recovered from both clinical specimens and food samples, in cereus II are emetic isolates and were shown to produce a functional emetic toxin peptide or were PCR positive for the emetic genetic determinants. The single ST 26 isolate that was negative for these emetic loci, E6345, was recovered from lung. It is possible this isolate never had these determinants or, since they are plasmid-borne [43], the plasmid may have been cured prior to or during laboratory manipulations. We have limited information on the case from which E6345 originated. It is possible that the pneumonia may have resulted from aspiration due to infection with bacteria that originally produced emetic toxin. The clade 1/Cereus II lineage appears to contain mostly isolates associated with severe systemic infections and emesis.

Several studies have suggested that *B. cereus *isolates that cluster closely with *B. anthracis *are more likely to be associated with clinical cases as opposed to environmental sources [4,44,45]. This suggests that it may be possible to infer the virulence of a strain (i.e., pathogenic potential) based on ST. The MLST data from this study suggests strains associated with severe disease presentations (pneumonia, septicemia, and bacteremia) are phylogenetically diverse within clades 1 and 2 but continue to appear restricted to these clades and are not present in clade 3. Thus, while clinical isolates are diverse, there may be differences in pathogenic potential at the clade level of resolution. In addition, some lineages appear to have more clinical isolates associated with severe or emetic illness such as the Cereus II and III lineages. Whether these or additional lineages or clonal complexes contain isolates primarily associated with specific diseases may become clearer as more clinical and environmental isolates are added to the database. Attempts to correlate ST with pathogenic potential of *B. cereus *are complicated by several factors. First, the pathogenic potential of environmental isolates is unknown. The source of an isolate (clinical versus environmental) does not necessarily correlate with pathogenic potential. *B. cereus *is not an obligate pathogen, therefore, isolates like those recovered from clinical specimens should also be found in the environment. Studies have shown that environmental isolates of the *B. cereus *group harbor a variety of *B. cereus *virulence genes, such as hemolysins, enterotoxins, cytotoxin K and phospholipase C [46,47]. Host susceptibility and whether or not *B. cereus *is associated with the primary infection or is a secondary infection may also complicate efforts to segregate strains or clonal complexes by virulence or pathogenic potential.

## Conclusion

Clinical isolates in general are diverse and well represented throughout clades 1 and 2, while no clinical isolates have been identified in clade 3. STs of isolates associated with severe disease were distributed throughout clades 1 and 2; however, the majority of GI isolates within clade 1 were limited to emetic strains of *B. cereus*. There was some evidence, based on limited numbers, of isolates within lineages or clonal complexes being associated with severe disease or a specific illness such as ST 78 with pneumonia. However, further work will be needed to clearly understand the relationship of isolates within the clades and lineages and how they may be related to the potential of isolates to cause a variety of illnesses. A more robust picture of the *B. cereus *group is not yet realized due to low number of sequenced isolates and biased sampling. The existence of multiple MLST schemes for the *B. cereus *group (n = 5) further hampers efforts. A recent report, however, describes the creation of a supertree database (SuperCAT) that combines and integrates data from all the published schemes [48] which should aid in the effort to better view the *B. cereus *group phylogeny. The true population structure of *B. cereus *will emerge as more diverse isolates, including clinical isolates with good epidemiological data, are analyzed and added to the public databases.

## Competing interests

The authors declare that they have no competing interests.

## Authors' contributions

ARH and PPW contributed to study design, analysis, and writing of the manuscript. RTN performed MLST and contributed to study design and writing of manuscript. CKM performed MLST and contributed to writing of manuscript. JEG performed MLST, data analysis and contributed to writing of manuscript. LH and JP contributed to study design and writing of manuscript. The manuscript has been reviewed and approved by all authors.

## References

[B1] Lechner S, Mayr R, Francis KP, Pruss BM, Kaplan T, Wiessner-Gunkel E, Stewart GS, Scherer S (1998). *Bacillus weihenstephanensis *sp. nov. is a new psychrotolerant species of the *Bacillus cereus *group. Int J Syst Bacteriol.

[B2] Jensen GB, Hansen BM, Eilenberg J, Mahillon J (2003). The hidden lifestyles of *Bacillus cereus *and relatives. Environ Microbiol.

[B3] Barker M, Thakker B, Priest FG (2005). Multilocus sequence typing reveals that *Bacillus cereus *strains isolated from clinical infections have distinct phylogenetic origins. FEMS Microbiol Lett.

[B4] Helgason E, Tourasse NJ, Meisal R, Caugant DA, Kolsto AB (2004). Multilocus sequence typing scheme for bacteria of the *Bacillus cereus *group. Appl Environ Microbiol.

[B5] Drobniewski FA (1993). *Bacillus cereus *and related species. Clin Microbiol Rev.

[B6] Kotiranta A, Lounatmaa K, Haapasalo M (2000). Epidemiology and pathogenesis of *Bacillus cereus *infections. Microbes Infect.

[B7] Bekemeyer WB, Zimmerman GA (1985). Life-threatening complications associated with *Bacillus cereus *pneumonia. Am Rev Respir Dis.

[B8] Coonrod JD, Leadley PJ, Eickhoff TC (1971). *Bacillus cereus *pneumonia and bacteremia. A case report. Am Rev Respir Dis.

[B9] Frankard J, Li R, Taccone F, Struelens MJ, Jacobs F, Kentos A (2004). *Bacillus cereus *pneumonia in a patient with acute lymphoblastic leukemia. Eur J Clin Microbiol Infect Dis.

[B10] Funada H, Machi T, Matsuda T (1991). *Bacillus cereus *pneumonia with empyema complicating aplastic anemia–a case report. Kansenshogaku Zasshi.

[B11] Gascoigne AD, Richards J, Gould K, Gibson GJ (1991). Successful treatment of *Bacillus cereus *infection with ciprofloxacin. Thorax.

[B12] Leff A, Jacobs R, Gooding V, Hauch J, Conte J, Stulbarg M (1977). *Bacillus cereus *pneumonia. Survival in a patient with cavitary disease treated with gentamicin. Am Rev Respir Dis.

[B13] Sliman R, Rehm S, Shlaes DM (1987). Serious infections caused by *Bacillus *species. Medicine (Baltimore).

[B14] Strauss R, Mueller A, Wehler M, Neureiter D, Fischer E, Gramatzki M, Hahn EG (2001). Pseudomembranous tracheobronchitis due to *Bacillus cereus*. Clin Infect Dis.

[B15] Jonsson S, Clarridge J, Young EJ (1983). Necrotizing pneumonia and empyema caused by *Bacillus cereus *and *Clostridium bifermentans*. Am Rev Respir Dis.

[B16] Miller JM, Hair JG, Hebert M, Hebert L, Roberts FK, Weyant RS (1997). Fulminating bacteremia and pneumonia due to *Bacillus cereus *. J Clin Microbiol.

[B17] Hoffmaster AR, Ravel J, Rasko DA, Chapman GD, Chute MD, Marston CK, De BK, Sacchi CT, Fitzgerald C, Mayer LW, Maiden M, Priest FG, Barker M, Jiang L, Cer RZ, Rilsonte J, Peterson SN, Weyant RS, Galloway DR, Read TD, Popovic T, Fraser CM (2004). Identification of anthrax toxin genes in a *Bacillus cereus *associated with an illness resembling inhalation anthrax. Proc Natl Acad Sci USA.

[B18] Avashia SB, Riggins WS, Lindley C, Hoffmaster A, Drumgoole R, Nekomoto T, Jackson PJ, Hill KK, Williams K, Lehman L, Libal MC, Wilkins PP, Alexander J, Tvaryanas A, Betz T (2007). Fatal pneumonia among metalworkers due to inhalation exposure to *Bacillus cereus *containing *Bacillus anthracis *toxin genes. Clin Infect Dis.

[B19] Rasko DA, Rosovitz MJ, Okstad OA, Fouts DE, Jiang L, Cer RZ, Kolsto AB, Gill SR, Ravel J (2007). Complete sequence analysis of novel plasmids from emetic and periodontal *Bacillus cereus *isolates reveals a common evolutionary history among the *B. cereus*-group plasmids, including *Bacillus anthracis *pXO1. J Bacteriol.

[B20] Pannucci J, Okinaka RT, Sabin R, Kuske CR (2002). *Bacillus anthracis *pXO1 plasmid sequence conservation among closely related bacterial species. J Bacteriol.

[B21] Pannucci J, Okinaka RT, Williams E, Sabin R, Ticknor LO, Kuske CR (2002). DNA sequence conservation between the *Bacillus anthracis *pXO2 plasmid and genomic sequence from closely related bacteria. BMC Genomics.

[B22] Hoffmaster AR, Hill KK, Gee JE, Marston CK, De BK, Popovic T, Sue D, Wilkins PP, Avashia SB, Drumgoole R, Helma H, Ticknor LO, Okinaka RT, Jackson PJ (2006). Characterization of *Bacillus cereus *isolates associated with fatal pneumonias: strains are closely related to *Bacillus anthracis *and harbor *B. anthracis *virulence genes. J Clin Microbiol.

[B23] Antonini JM (2003). Health effects of welding. Crit Rev Toxicol.

[B24] Coggon D, Inskip H, Winter P, Pannett B (1994). Lobar pneumonia: an occupational disease in welders. Lancet.

[B25] Doig AT, Challen PJ (1964). Respiratory hazards in welding. Ann Occup Hyg.

[B26] Smith JM, Smith NH, O'Rourke M, Spratt BG (1993). How clonal are bacteria?. Proc Natl Acad Sci USA.

[B27] Sorokin A, Candelon B, Guilloux K, Galleron N, Wackerow-Kouzova N, Ehrlich SD, Bourguet D, Sanchis V (2006). Multiple-locus sequence typing analysis of *Bacillus cereus *and *Bacillus thuringiensis *reveals separate clustering and a distinct population structure of psychrotrophic strains. Appl Environ Microbiol.

[B28] Priest FG, Barker M, Baillie LW, Holmes EC, Maiden MC (2004). Population structure and evolution of the *Bacillus cereus *group. J Bacteriol.

[B29] Ehling-Schulz M, Svensson B, Guinebretiere MH, Lindback T, Andersson M, Schulz A, Fricker M, Christiansson A, Granum PE, Martlbauer E, Nguyen-The C, Salkinoja-Salonen M, Scherer S (2005). Emetic toxin formation of *Bacillus cereus *is restricted to a single evolutionary lineage of closely related strains. Microbiology.

[B30] Helgason E, Caugant DA, Olsen I, Kolsto AB (2000). Genetic structure of population of *Bacillus cereus *and *B. thuringiensis *isolates associated with periodontitis and other human infections. J Clin Microbiol.

[B31] Tourasse NJ, Helgason E, Okstad OA, Hegna IK, Kolsto AB (2006). The *Bacillus cereus *group: novel aspects of population structure and genome dynamics. J Appl Microbiol.

[B32] Vassileva M, Torii K, Oshimoto M, Okamoto A, Agata N, Yamada K, Hasegawa T, Ohta M (2006). Phylogenetic analysis of *Bacillus cereus *isolates from severe systemic infections using multilocus sequence typing scheme. Microbiol Immunol.

[B33] Sacchi CT, Whitney AM, Mayer LW, Morey R, Steigerwalt A, Boras A, Weyant RS, Popovic T (2002). Sequencing of 16S rRNA gene: a rapid tool for identification of *Bacillus anthracis *. Emerg Infect Dis.

[B34] Hoffmaster AR, Fitzgerald CC, Ribot E, Mayer LW, Popovic T (2002). Molecular subtyping of *Bacillus anthracis *and the 2001 bioterrorism-associated anthrax outbreak, United States. Emerg Infect Dis.

[B35] Ehling-Schulz M, Fricker M, Scherer S (2004). Identification of emetic toxin producing *Bacillus cereus *strains by a novel molecular assay. FEMS Microbiol Lett.

[B36] Marston CK, Gee JE, Popovic T, Hoffmaster AR (2006). Molecular approaches to identify and differentiate *Bacillus anthracis *from phenotypically similar *Bacillus *species isolates. BMC Microbiol.

[B37] Saitou N, Nei M (1987). The neighbor-joining method: a new method for reconstructing phylogenetic trees. Mol Biol Evol.

[B38] Kimura M (1980). A simple method for estimating evolutionary rates of base substitutions through comparative studies of nucleotide sequences. J Mol Evol.

[B39] Kumar S, Tamura K, Nei M (2004). MEGA3: Integrated software for Molecular Evolutionary Genetics Analysis and sequence alignment. Brief Bioinform.

[B40] Hunter PR, Gaston MA (1988). Numerical index of the discriminatory ability of typing systems: an application of Simpson's index of diversity. J Clin Microbiol.

[B41] Vassileva M, Torii K, Oshimoto M, Okamoto A, Agata N, Yamada K, Hasegawa T, Ohta M (2007). A new phylogenetic cluster of cereulide-producing *Bacillus cereus *strains. J Clin Microbiol.

[B42] Sue D, Hoffmaster AR, Popovic T, Wilkins PP (2006). Capsule production in *Bacillus cereus *strains associated with severe pneumonia. J Clin Microbiol.

[B43] Hoton FM, Andrup L, Swiecicka I, Mahillon J (2005). The cereulide genetic determinants of emetic *Bacillus cereus *are plasmid-borne. Microbiology.

[B44] Helgason E, Okstad OA, Caugant DA, Johansen HA, Fouet A, Mock M, Hegna I, Kolsto (2000). *Bacillus anthracis*, *Bacillus cereus*, and *Bacillus thuringiensis*–one species on the basis of genetic evidence. Appl Environ Microbiol.

[B45] Hill KK, Ticknor LO, Okinaka RT, Asay M, Blair H, Bliss KA, Laker M, Pardington PE, Richardson AP, Tonks M, Beecher DJ, Kemp JD, Kolsto A, Lee Wong AC, Keim P, Jackson PJ (2004). Fluorescent amplified fragment length polymorphism analysis of *Bacillus anthracis*, *Bacillus cereus*, and *Bacillus thuringiensis *isolates. Appl Environ Microbiol.

[B46] Hendriksen NB, Hansen BM, Johansen JE (2006). Occurrence and pathogenic potential of *Bacillus cereus *group bacteria in a sandy loam. Antonie Van Leeuwenhoek.

[B47] Moravek M, Dietrich R, Buerk C, Broussolle V, Guinebretiere MH, Granum PE, Nguyen-The C, Martlbauer E (2006). Determination of the toxic potential of *Bacillus cereus *isolates by quantitative enterotoxin analyses. FEMS Microbiol Lett.

[B48] Tourasse NJ, Kolsto AB (2008). SuperCAT: a supertree database for combined and integrative multilocus sequence typing analysis of the *Bacillus cereus *group of bacteria (including *B. cereus*, *B. anthracis *and *B. thuringiensis*). Nucleic Acids Res.

